# Diagnostic Approach to Secondary Hypertension: Atherosclerotic Renal Artery Stenosis

**DOI:** 10.7759/cureus.101104

**Published:** 2026-01-08

**Authors:** André Filipe Conchinha, Tiago Pack, Afonso Rodrigues, Sofia Cunha, Pedro Santos

**Affiliations:** 1 Internal Medicine, Centro Hospitalar Universitário de Lisboa Central, Lisbon, PRT; 2 Medicine, Centro Hospitalar Universitário de Lisboa Central, Lisbon, PRT; 3 Internal Medicine, Centro Hospitalar do Médio Tejo, Abrantes, PRT

**Keywords:** atherosclerosis, atherosclerotic renal artery stenosis, chronic kidney disease (ckd), secondary hypertension, treatment-resistant hypertension

## Abstract

Secondary hypertension stems from identifiable causes, making targeted treatment essential to control blood pressure (BP) and mitigate the occurrence of complications.

We describe a case of resistant hypertension in a 48-year-old male referred to the internal medicine clinic for investigation. A complementary study with abdominal and pelvic computed tomography revealed significant aortic atherosclerosis involving the emergence of the renal arteries, with particularly evident stenosis on the right. Following vascular intervention, the patient's BP stabilized with just two antihypertensive agents at reduced doses.

This case illustrates how extensive renal artery atherosclerosis can cause significant stenosis, leading to secondary hypertension.

## Introduction

Secondary hypertension is defined as elevated blood pressure resulting from an identifiable, often treatable, underlying cause. It accounts for approximately 5-10% of adult hypertension cases. The American College of Cardiology and American Heart Association recommend considering secondary hypertension in patients with stage 2 hypertension, treatment-resistant hypertension, sudden onset or worsening of previously controlled hypertension, early-onset hypertension (<30 years), and those with disproportionate target organ damage. The main causes include renal parenchymal disease, stenosis of one or both renal arteries, primary hyperaldosteronism, phaeochromocytoma, Cushing's syndrome, hyper- or hypothyroidism, obstructive sleep apnea (OSA), coarctation of the aorta, and ingestion of certain substances. The rationale for identifying secondary causes is that targeted treatment of the underlying condition can result in significant improvement in blood pressure control and reduction in cardiovascular risk [1,2].

In young adults, the most frequent causes are primary aldosteronism, renovascular hypertension (including fibromuscular dysplasia), and primary kidney disease. Notably, a large cross-sectional study found that among hypertensive patients aged 18-40 years, 29.6% had a secondary cause, with primary aldosteronism accounting for over half of these cases, followed by renovascular hypertension and kidney disease. In children, renal parenchymal disease and coarctation of the aorta predominate [2-5].

In middle-aged and older adults, the spectrum shifts; chronic kidney disease, atherosclerotic renal artery stenosis (ARAS), and OSA become increasingly common, while endocrine causes such as pheochromocytoma and Cushing syndrome remain rare but clinically important due to their potential for cure with targeted therapy. Drug-induced hypertension is also a significant contributor, with agents such as nonsteroidal anti-inflammatory drugs, oral contraceptives, sympathomimetics, and certain antidepressants implicated in raising blood pressure [5-8].

Rare causes, such as vascular anomalies (e.g., neurofibromatosis type 1, arteritis, and aortic coarctation), and thrombotic or embolic renal artery lesions, should be considered in select cases, especially when initial workup is unrevealing or clinical features are atypical [9].

This case illustrates secondary hypertension due to ARAS in a middle-aged adult (>40 years old), which is consistent with the etiologies described for each age group.

## Case presentation

The patient was a 48-year-old male, independent in activities of daily living. He had a history of arterial hypertension and stage 2 chronic kidney disease. He was referred for an internal medicine consultation due to poorly controlled hypertension (systolic blood pressure of 150-170 mmHg and diastolic blood pressure of 80-100 mmHg), while on four classes of antihypertensives (amlodipine 10 mg per day, candesartan 32 mg per day, bisoprolol 5 mg 12 hourly, hydrochlorothiazide 150 mg per day, and isosorbide dinitrate 20 mg 12 hourly).

Considering the history of hypertension diagnosed at an early age (around 43 years old) and the persistence of resistant hypertension, a study of secondary hypertension was performed. Blood levels of sodium, potassium, renin, and aldosterone were within normal limits. Thyroid function was also normal. On physical examination, the patient did not have signs suggestive of Cushing's syndrome, but free cortisol in 24-hour urine was measured and showed no alterations (<120 micrograms). The Epworth Sleepiness Scale was not suggestive of OSA (Table 1).

**Table 1:** Analytical values ​​in the context of studying the causes of secondary hypertension. GFR: glomerular filtration rate; TSH: thyroid-stimulating hormone; T4: thyroxine hormone.

Test​	Value​	Unit​	Reference value​
Hemoglobin​	14.3​	g/dl​	13.5 – 17​
Creatinine​	1.4​	mg/dl​	<1.1​
GFR​	63​	ml/min/1.73 m²​	>90​
Sodium​	141​	mEq/L​	135 – 145​
Potassium​	3.8​	mEq/L​	3.5 – 5​
Renin​	16.3​	pg/ml​	2.4 – 22​
Aldosterone​	6.2​	ng/dl​	14 – 15​
TSH​	0.339​	mlU/L​	0.3 – 0.4
T4​	1.24​	ng/dl​	0.7 – 1.8​

**Figure 1:**
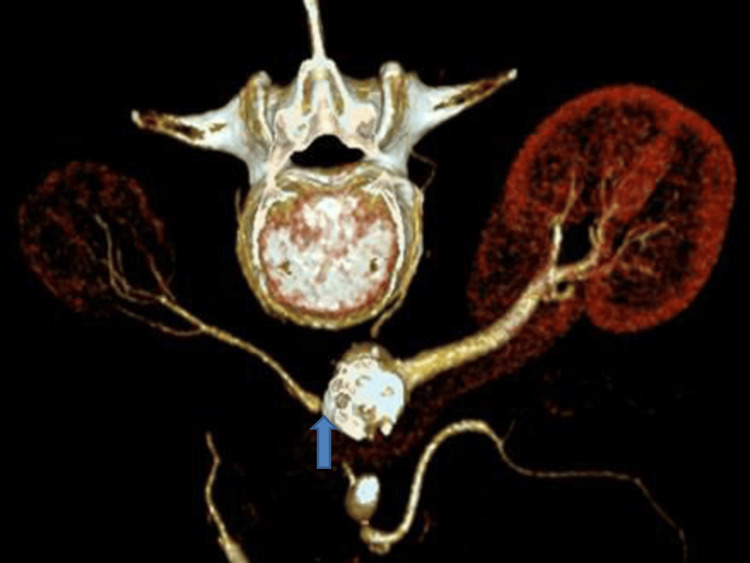
3D reconstruction of abdominal and pelvic axial CT scans. The arrow indicates the origin of the right renal artery, with significant aortic atherosclerosis and consequent atrophy of the ipsilateral kidney.

**Figure 2:**
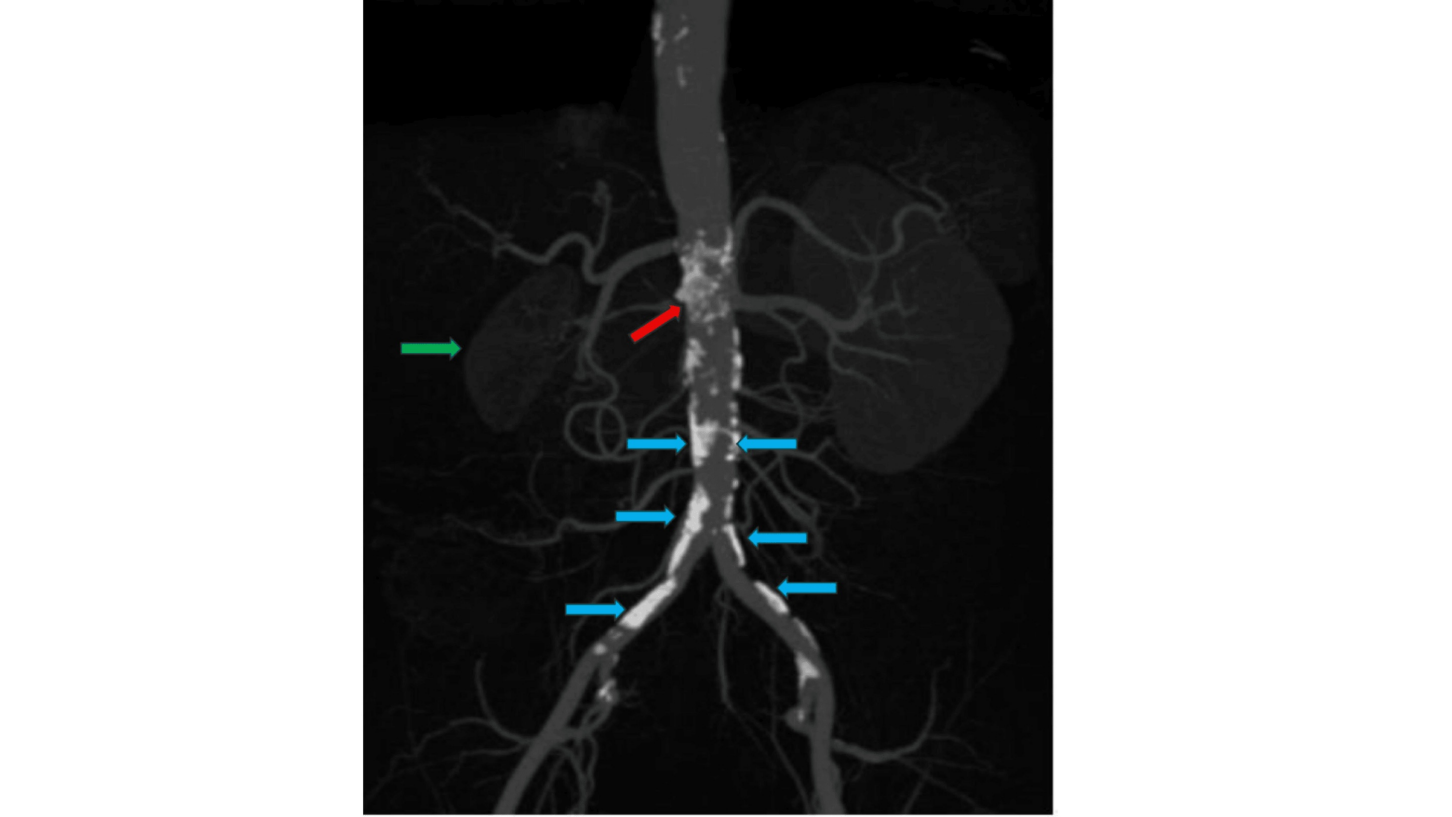
Abdominal and pelvic CT scan in frontal section. Red arrow: Ostium of the right renal artery surrounded by atherosclerotic plaque. Green arrow: Atrophic right kidney. Blue arrows: Multiple atherosclerotic plaques.

Doppler ultrasound revealed bilateral stenosis, with 95% obstruction on the right and 70% on the left. An abdominal and pelvic CT scan documented the following findings: “Atherosclerotic disease of the abdominal aortic wall at the origin of the renal arteries, with apparent involvement of the ostium of the right renal artery, as well as ipsilateral renal atrophy, suggesting a renovascular etiology” (Figures 1, 2).

The remainder of the analytical study reported creatinine of 1.4 mg/dl with a glomerular filtration rate of 63 ml/min/1.73 m², total cholesterol (TC) of 253 mg/dl, and low-density lipoprotein (LDL) cholesterol of 181 mg/dl. Considering the cardiovascular risk according to the Systematic Coronary Risk Evaluation 2 (SCORE2), the patient presents a high risk (hypertension and chronic kidney disease); therefore, the LDL target should be <70 mg/dl, having initiated therapy with rosuvastatin 20 mg and ezetimibe 10 mg, with simultaneous indication for dietary care and regular physical activity [10]. He also underwent coronary angio-CT, which documented atherosclerotic plaques without hemodynamic significance (50% occlusion in the right coronary artery and 30% in the circumflex artery), an echocardiogram with good global systolic function, and Doppler ultrasound of the neck vessels without significant changes.

The case was discussed with the vascular surgery department, and a right renal artery angioplasty with stent was indicated. He also started antiplatelet therapy with acetylsalicylic acid and clopidogrel. Three months after surgery, the patient achieved adequate blood pressure control on only two antihypertensive drugs (amlodipine 10 mg per day and candesartan 8 mg per day). Regarding the lipid profile, three months after starting therapy and lifestyle changes, total cholesterol (TC) was 187 mg/dl, and LDL was 76 mg/dl (reduction > 50% and close to the target of 70 mg/dl) (Table 2).

**Table 2:** Evolution of the patient's lipid profile after three months of treatment. T​C: total cholesterol; LDL: low-density lipoprotein cholesterol; HDL: high-density lipoprotein cholesterol; TG: triglycerides.

Test​	1st day​	90th day​	Unit​	Reference value​
T​C	253​	187​	mg/dl​	<190​
LDL​	181​	76​	mg/dl​	<70​
HDL​	53​	61​	mg/dl​	>40​
TG​	151​	117​	mg/dl​	<150​

## Discussion

Secondary hypertension arises from an underlying clinical condition and accounts for 5% to 10% of all cases of hypertension. The identification and treatment of the underlying cause is crucial, as this can lead to the normalization of blood pressure, thereby mitigating the risk of cardiovascular complications [1,11].

According to the American College of Cardiology and the American Heart Association, atherosclerotic disease accounts for approximately 90% of cases of renal artery stenosis, but only a small fraction (0.1%-5%) of these cases are hemodynamically significant enough to result in renovascular hypertension. The presence of ARAS indicates systemic vascular disease and is associated with increased risk for kidney failure and cardiovascular morbidity [1,12].

The first-line management for hypertension due to ARAS is optimal medical therapy, which includes antihypertensive agents (preferably a renin-angiotensin system blocker), high-intensity statin therapy, smoking cessation, glycemic control in diabetes, and antiplatelet therapy. This recommendation is based on randomized controlled trials, which found no clear advantage of revascularization (angioplasty or stenting) over medical therapy in most patients [1,12]. Revascularization may be considered only in select patients with uncontrolled hypertension and either progressive kidney dysfunction or recurrent, sudden-onset pulmonary edema, as these subgroups were not well represented in clinical trials and may benefit from intervention [1]. The rationale for this approach is that aggressive medical therapy effectively reduces cardiovascular risk and blood pressure in most cases, while procedural intervention carries additional risks and has not shown superior outcomes in the general population with atherosclerotic renal artery stenosis. In this case, the vascular surgery team considered proceeding with angioplasty, deeming this intervention necessary for adequate blood pressure control. This decision was based on insufficient blood pressure control (already for several months with five classes of antihypertensive drugs) and the significant degree of right-sided stenosis with renal atrophy [1,12,13].

ARAS is the predominant etiology of renovascular hypertension in adults, accounting for over 90% of cases, particularly in those over 65 years of age and in patients with established atherosclerotic disease elsewhere (coronary, carotid, or peripheral arteries). In the case presented, the patient was under 50 years old and had no known atherosclerosis in other areas. However, he presented with an altered lipid profile, and further investigation showed generalized atherosclerosis.

Clinically, ARAS should be suspected in patients with an abrupt onset or worsening hypertension after age 55, resistant hypertension, unexplained renal dysfunction, or recurrent flash pulmonary edema. Physical findings such as an abdominal bruit and imaging evidence of asymmetric renal size further support the diagnosis. The pathophysiology involves activation of the renin-angiotensin-aldosterone system due to reduced renal perfusion, leading to secondary hypertension that may be difficult to control with standard antihypertensive regimens [12-14]. Noninvasive imaging modalities, including duplex ultrasonography, CT angiography, and magnetic resonance angiography, are preferred for diagnosis, with angiography reserved for select cases [15,16].

The overall approach emphasizes individualized assessment and risk factor modification, as ARAS is a marker of systemic vascular disease and increased cardiovascular risk [12-16].

## Conclusions

Secondary hypertension represents a minority of hypertensive patients; therefore, it is essential to maintain a high level of suspicion regarding certain factors. Etiological diagnosis and corresponding treatment are essential to achieve adequate blood pressure control.

This article highlights the role of atherosclerosis in the pathogenesis of secondary hypertension due to renovascular causes. The case described, with significant ARAS, required a multidisciplinary approach with vascular surgery intervention to achieve adequate blood pressure control.
